# Supplementary material for: Ribosomal stalling landscapes revealed by high-throughput inverse toeprinting of mRNA libraries
Source: Life Sci Alliance. 2018 Oct 9;1(5):e201800148. doi: 10.26508/lsa.201800148 (PMC6238534; doi:10.26508/lsa.201800148)
Supplement: Supplementary file 1 [file LSA-2018-00148_TableS1.docx]

**Supplementary Table S1 – Observed and possible variants in the ErmBL library**

|  | DNA | | | Protein | | |
| --- | --- | --- | --- | --- | --- | --- |
| **Mutations** | **Observed** | **Possible** | **Percentage** | **Observed** | **Possible*** | **Percentage** |
| 0 | 1 | 1 | 100 | 1 | 1 | 100 |
| 1 | 90 | 90 | 100 | 190 | 190 | 100 |
| 2 | 3,915 | 3,915 | 100 | 8,975 | 16,245 | 55.3 |
| 3 | 76,844 | 109,620 | 70.1 | 81,760 | 823,080 | 9.9 |
| * Does not include stop codons | | | | | | |
