# Supplementary material for: Ribosomal stalling landscapes revealed by high-throughput inverse toeprinting of mRNA libraries
Source: Life Sci Alliance. 2018 Oct 9;1(5):e201800148. doi: 10.26508/lsa.201800148 (PMC6238534; doi:10.26508/lsa.201800148)
Supplement: Supplementary file 2 [file LSA-2018-00148_TableS2.docx]

**Supplementary Table S2 – ErmBL variants that discriminate between Ery and Ole**

| ErmBL variant | log2(*F*_Ery_/*F*_Ole_) | Independent DNA sequences |
| --- | --- | --- |
| **Single mutants** | | |
| MLVFQMLNVDK | +1.95 | 198 |
| MLVFQMCNVDK | +1.62 | 90 |
| MLVFQM**P**NVDK | +1.44 | 83 |
| MLVFQMRNVD**T** | +1.23 | 97 |
| MLVF**R**MRNVDK | +1.16 | 144 |
| MLVFQMRNVD**I** | +1.04 | 226 |
| ML**F**FQMRNVDK | +1.01 | 48 |
| **Double mutants** | | |
| MLVFHMLNVDK | +2.68 | 72 |
| MLVLQMLNVDK | +2.22 | 83 |
| MLVFQMLKVDK | +1.91 | 55 |
| MLVLQMRNVDN | +1.82 | 94 |
| MLVLQMRNVDI | +1.80 | 106 |
| MFVFQMRNVDI | +1.66 | 54 |
| MLVFHMRNVDN | +1.65 | 125 |
| MLVFQIRNVDN | +1.56 | 137 |
| MLVFQIRNVDI | +1.56 | 162 |
| MLAFQMLNVDK | +1.51 | 46 |
| MSVFQMRNVDI | +1.49 | 60 |
| MLVFQMRKLDK | +1.41 | 70 |
| MLVFLMRNVDN | +1.41 | 80 |
| MLVFKIRNVDK | +1.34 | 80 |
| MLVFQMRKVDN | +1.32 | 95 |
| MLLFQRRNVDK | +1.29 | 82 |
| MLVFPTRNVDK | +1.28 | 79 |
| MLVFQIRNVDE | +1.18 | 63 |
| MLLFPMRNVDK | +1.17 | 112 |
| MLLFQMRKVDK | +1.16 | 124 |
| MLVFHMRNVDI | +1.15 | 115 |
| MLVFQVRNVDI | +1.14 | 62 |
| MMVLQMRNVDK | +1.12 | 86 |
| MLVFQMLTVDK | +1.09 | 58 |
| MLLFEMRNVDK | +1.08 | 70 |
| MLVFQMLIVDK | +1.08 | 74 |
| MLVFHMRNVGK | +1.04 | 71 |
| MFVFLMRNVDK | –1.03 | 90* |
| Variants with a combined 150 reads between the Ery and Ole samples that were enriched at least 2-fold upon inverse toeprinting in the presence of one antibiotic but not the other are shown here. Only single and double mutants are shown, with the mutated residues underlined. The number of individual DNA variants in the Ery sample that gave rise to these variants is indicated. For the sample marked with *, the number of individual DNA variants in the Ole sample is given. | | |
