# Supplementary material for: Ribosomal stalling landscapes revealed by high-throughput inverse toeprinting of mRNA libraries
Source: Life Sci Alliance. 2018 Oct 9;1(5):e201800148. doi: 10.26508/lsa.201800148 (PMC6238534; doi:10.26508/lsa.201800148)
Supplement: Supplementary file 3 [file LSA-2018-00148_TableS3.docx]

**Supplementary Table S3 – Oligonucleotides used for inverse toeprinting**

| Number | New name | Sequence 5’-3’ | Comments | Supplier |
| --- | --- | --- | --- | --- |
| 1 | T7_RBS_ATG_f | CGA-TCG-AAT-TCT-AAT-ACG-ACT-CAC-TAT-AGG-GCT-TAA-GTA-TAA-GGA-GGA-AAA-AAT-ATG | DNA template generation | Eurogentec |
| 2 | Stop_EcoRV_r | TAT-ATG-GAT-CCT-TTT-TGA-TAT-TGA-TAT-CTC-ATC-ACA-CCG-AGA-TCG | DNA template generation | Eurogentec |
| 3 | T7_f | CGA-TCG-AAT-TCT-AAT-ACG-ACT-CAC-TAT-AG | DNA template generation | Eurogentec |
| 4 | EcoRV_r | TAT-ATG-GAT-CCT-TTT-TGA-TAT-TGA-TA | DNA template generation | Eurogentec |
| 5 | ermAL1_template | GGA-GGA-AAA-AAT-ATG-TGC-ACC-AGT-ATC-GCA-GTA-GTA-GAA-ATT-ACT-TTA-TCT-CAT-GCG-ATC-TCG-GTG-TAA-T | ermAL1 WT template | Eurogentec |
| 6 | ermBL_template | GGA-GGA-AAA-AAT-ATG-TTG-GTA-TTC-CAA-ATG-CGT-AAT-GTA-GAT-AAA-ACA-TCT-ACT-ATT-TTG-AAA-GCG-ATC-TCG-GTG-TAA-T | ermBL WT template | Eurogentec |
| 7 | ermCL_template | GGA-GGA-AAA-AAT-ATG-GGC-ATT-TTT-AGT-ATT-TTT-GTA-ATC-AGC-GCG-ATC-TCG-GTG-TAA-T | ermCL WT template | Eurogentec |
| 8 | ermDL_template | GGA-GGA-AAA-AAT-ATG-ACA-CAC-TCA-ATG-AGA-CTT-CGT-TTC-CCA-ATT-ACT-TTG-AAC-CAG-GCG-ATC-TCG-GTG-TAA-T | ermDL WT template | Eurogentec |
| 9 | secM_template | GAG-GAA-AAA-ATA-TGT-TCA-GCA-CGC-CCG-TCT-GGA-TAA-GCC-AGG-CGC-AAG-GCA-TCC-GTG-CTG-GCC-CTG-CGA-TCT-CGG-TGT-A | secM WT template | Eurogentec |
| 10 | tnaC_template | GGA-GGA-AAA-AAT-ATG-TGG-TTC-AAT-ATT-GAC-AAC-AAA-ATT-GTC-GAT-CAC-CGC-CCT-TAA-GCG-ATC-TCG-GTG-TAA-T | tnaC template | Eurogentec |
| 11 | ermBL_deep_mutated | GGA-GGA-AAA-AAT-ATG-**TTG-GTA-TTC-CAA-ATG-CGT-AAT-GTA-GAT-AAA-**GCG-ATC-TCG-GTG-TGA-T | ermBL library,  79% of the wild-type nucleotide and 7% of each of the three other types of nucleotide was added at each position in bold | Eurofins |
| 12 | NNS15 | GGA-GGA-AAA-AAT-ATG-**NNS-NNS-NNS-NNS-NNS-NNS-NNS-NNS-NNS-NNS-NNS-NNS-NNS-NNS-NNS**-GCG-ATC-TCG-GTG-TAA | NNS_15_ library template, 25% of each nucleotide was added at each N position and 50% of C or G were added at each S position | Eurogentec |
| 13 | 3’_linker_EcoRV | /5rAPP/GGT-ATC-TCG-GTG-TGA-CTG-ACT-GAG-ATA-TCC-TGT-AGG-CAC-CAT-CAA-T/ddC | linker encoding *EcoRV* restriction site | IDT |
| 14 | 3’_linker_ApoI | /5rAPP/GGT-ATC-TCG-GTG-TGA-CTG-ACT-GAA-AAT-TTC-TGT-AGG-CAC-CAT-CAA-T/ddC | linker encoding *ApoI* restriction site | IDT |
| 15 | Linker_r | ATT-GAT-GGT-GCC-TAC-AG | reverse transcription | Eurogentec |
| 16 | cDNA_f | GTA-TAA-GGA-GGA-AAA-AAT-ATG | cDNA amplification | Eurogentec |
| 17 | Biotin_standard | /5Biosg/AAA-AAA-AAA-AAA-AAT-TAA-CTC-CAT-CTA-A | Chemical biotinylation on the 5’-end | IDT |
