# Supplementary material for: Ribosomal stalling landscapes revealed by high-throughput inverse toeprinting of mRNA libraries
Source: Life Sci Alliance. 2018 Oct 9;1(5):e201800148. doi: 10.26508/lsa.201800148 (PMC6238534; doi:10.26508/lsa.201800148)
Supplement: Supplementary file 4 [file LSA-2018-00148_TableS4.docx]

**Supplementary Table S4 – Oligonucleotides used for generating the Illumina sequencing library and barcoding**

| Number | New Name | Sequence 5’-3’ | Comments | Supplier |
| --- | --- | --- | --- | --- |
| 18 | NGS_f | AAT-GAT-ACG-GCG-ACC-ACC-G | short amplifying oligo | Eurogentec |
| 19 | NGS_r | CAA-GCA-GAA-GAC-GGC-ATA-CGA-G | short amplifying oligo | Eurogentec |
| 20 | NGS_adapter_f | AAT-GAT-ACG-GCG-ACC-ACC-GAG-ATC-TAC-ACT-CTT-TCC-CTA-CAC-GAC-GCT-CTT-CCG-ATC-TGT-ATA-AGG-AGG-AAA-AAA-TAT-G | NGS fwd oligo | Eurogentec |
| 21 | NGS_adapter_index1 | CAA-GCA-GAA-GAC-GGC-ATA-CGA-GAT-**CGT-GAT**-GTG-ACT-GGA-GTT-CAG-ACG-TGT-GCT-CTT-CCG-ATC-GAT-TGA-TGG-TGC-CTA-CAG | NNS_15_-NoAb1 | Eurogentec |
| 22 | NGS_adaper_index2 | CAA-GCA-GAA-GAC-GGC-ATA-CGA-GAT-**ACA-TCG**-GTG-ACT-GGA-GTT-CAG-ACG-TGT-GCT-CTT-CCG-ATC-GAT-TGA-TGG-TGC-CTA-CAG | NNS_15_-NoAb2  ErmBL-Ery | Eurogentec |
| 23 | NGS_adapter_index3 | CAA-GCA-GAA-GAC-GGC-ATA-CGA-GAT-**GCC-TAA**-GTG-ACT-GGA-GTT-CAG-ACG-TGT-GCT-CTT-CCG-ATC-GAT-TGA-TGG-TGC-CTA-CAG | NNS_15_-Ery1 | Eurogentec |
| 24 | NGS_adapter_index4 | CAA-GCA-GAA-GAC-GGC-ATA-CGA-GAT-**TGG-TCA**-GTG-ACT-GGA-GTT-CAG-ACG-TGT-GCT-CTT-CCG-ATC-GAT-TGA-TGG-TGC-CTA-CAG | NNS_15_-Ery2  ErmBL-Ole | Eurogentec |
| 25 | NGS_adapter_index13 | CAA-GCA-GAA-GAC-GGC-ATA-CGA-GAT-**TGT-TGA**-CTG-TGA-CTG-GAG-TTC-AGA-CGT-GTG-CTC-TTC-CGA-TCG-ATT-GAT-GGT-GCC-TAC-AG | NNS_15_-EF-P | Eurogentec |
| 26 | NGS_adapter_index14 | CAA-GCA-GAA-GAC-GGC-ATA-CGA-GAT-**ACG-GAA**-CTG-TGA-CTG-GAG-TTC-AGA-CGT-GTG-CTC-TTC-CGA-TCG-ATT-GAT-GGT-GCC-TAC-AG | NNS_15_-Ery + EF-P | Eurogentec |
| 27 | NGS_adapter_index15 | CAA-GCA-GAA-GAC-GGC-ATA-CGA-GAT-TC**T-GAC-AT**G-TGA-CTG-GAG-TTC-AGA-CGT-GTG-CTC-TTC-CGA-TCT-CAT-CAC-ACC-GAG-ATC-GC | ErmBL library | Eurogentec |
| 28 | NGS_adapter_index33 | CAA-GCA-GAA-GAC-GGC-ATA-CGA-GAT-**CGC-CTG**-GTG-ACT-GGA-GTT-CAG-ACG-TGT-GCT-CTT-CCG-ATC-TCA-TCA-CAC-CGA-GAT-CGC | NNS_15_ library | Eurogentec |
