# Supplementary material for: Ribosomal stalling landscapes revealed by high-throughput inverse toeprinting of mRNA libraries
Source: Life Sci Alliance. 2018 Oct 9;1(5):e201800148. doi: 10.26508/lsa.201800148 (PMC6238534; doi:10.26508/lsa.201800148)
Supplement: Supplementary file 5 [file LSA-2018-00148_TableS5.docx]

**Supplementary Table S5 – Oligonucleotides used for toeprinting**

| Number | New Name | Sequence 5’-3’ | Comments | Supplier |
| --- | --- | --- | --- | --- |
| 29 | TP_3’_spacer_r | CTT-GCC-TGC-GCA-CGA-AGA-GTA-CGG-ATG-TTG-TTC-AGA-GTC-AGT-TAT-TAT-TCG-CT | DNA template generation | Eurogentec |
| 30 | TP_NV1_r | GGT-TAT-AAT-GAA-TTT-TGC-TTA-TTA-ACC-TTG-CCT-GCG-CAC-G | DNA template generation | Eurogentec |
| 31 | TP_NV1_r_short | GGT-TAT-AAT-GAA-TTT-TGC-TT | DNA template generation | Eurogentec |
| 32 | TP_ermBL | GGA-GGA-AAA-AAT-ATG-TTG-GTA-TTC-CAA-ATG-CGT-AAT-GTA-GAT-AAA-ACA-TCT-ACT-ATT-TTG-AAA-AGC-GAA-TAA-TAA-CTG-ACT-CTG | ermBL WT toeprint template | Eurogentec |
| 33 | TP_ermBL_L7_f | GGA-GGA-AAA-AAT-ATG-TTG-GTA-TTC-CAA-ATG-CTT-AAT-GTA-GAT-AAA-ACA-TCT-ACT-ATT-TTG-AAA-AGC-GAA-TAA-TAA-CTG-ACT-CTG | ermBL L7 toeprint template | Eurogentec |
| 34 | TP_ermBL_L7K8_f | GGA-GGA-AAA-AAT-ATG-TTG-GTA-TTC-CAA-ATG-CTT-AAA-GTA-GAT-AAA-ACA-TCT | ermBL L7K8 toeprint template | Eurogentec |
| 35 | TP_ermBL_L7K8_r | CAG-AGT-CAG-TTA-TTA-TTC-GCT-TTT-CAA-AAT-AGT-AGA-TGT-TTT-ATC-TAC | ermBL L7 and L7K8 toeprint template | Eurogentec |
| 36 | NV1_YY (Vazquez-Laslop et al., 2008) | GGT-TAT-AAT-GAA-TTT-TGC-TTA-TTA-AC | Yakima Yellow labeled probe | Eurogentec |
