# Supplementary material for: Ribosomal stalling landscapes revealed by high-throughput inverse toeprinting of mRNA libraries
Source: Life Sci Alliance. 2018 Oct 9;1(5):e201800148. doi: 10.26508/lsa.201800148 (PMC6238534; doi:10.26508/lsa.201800148)
Supplement: Supplementary file 6 [file LSA-2018-00148_TableS6.docx]

**Supplementary Table S6 – Oligonucleotides used for generating the plasmids for *in vivo* studies**

| Number | New Name | Sequence 5’-3’ | Comments | Supplier |
| --- | --- | --- | --- | --- |
| 37 | pZa_f | ATG-TTT-TCC-TCC-TTA-TAA-GAC-TTA-ATT-AAG | Amplification of the plasmid backbone | Eurogentec |
| 38 | pZa_r | GTG-GTT-ATA-ATG-AAT-CGT-TAA-TAA-G | Amplification of the plasmid backbone | Eurogentec |
| 39 | pZa_ermBL_WT_f | GGA-GGA-AAA-CAT-ATG-TTG-GTA-TTC-CAA-ATG-CGT-AAT-GTA-G | Insert ermBL-CL hybrid | Eurogentec |
| 40 | pZa_ermBL_WT_r | TGG-TTG-ATA-ATG-AAC-TGT-TTT-ATC-TAC-ATT-ACG-CAT-TTG | Insert ermBL-CL hybrid | Eurogentec |
| 41 | pZa_ermBL_L7_f | CAT-ATG-TTG-GTA-TTC-CAA-ATG-CTT-AAT-GTA-GAT-AAA-ACA-GTT-CAT-TAT-CAA-CC | Point mutation of ermBL L7K8 to ermBL L7 | Eurogentec |
| 42 | pZa_ermBL_L7_r | GGT-TGA-TAA-TGA-ACT-GTT-TTA-TCT-ACA-TTA-AGC-ATT-TGG-AAT-ACC-AAC-ATA-TG | Point mutation of ermBL L7K8 to ermBL L7 | Eurogentec |
| 43 | pZa_ermBL_L7K8_f | GGA-GGA-AAA-CAT-ATG-TTG-GTA-TTC-CAA-ATG-CTT-AAA-GTA-G | Insert ermBL_L7K8-CL hybrid | Eurogentec |
| 44 | pZa_ermBL_L7K8_r | TGG-TTG-ATA-ATG-AAC-TGT-TTT-ATC-TAC-TTT-AAG-CAT-TTG | Insert ermBL_L7K8-CL hybrid | Eurogentec |
| 45 | pZa_MAKPW_f | GGA-GGA-AAA-CAT-ATG-GCC-AAG-CCC-TGG-ACA-GTT-CAT-TAT-CAA | Insert MAKPW-CL hybrid | Eurogentec |
| 46 | pZa_MAGPW_f | GGA-GGA-AAA-CAT-ATG-GCC-GGC-CCG-TGG-ACA-GTT-CAT-TAT-CAA | Insert MAGPW-CL hybrid | Eurogentec |
| 47 | pZa_MAPTW_f | CTT-ATA-AGG-AGG-AAA-ACA-TAT-GGC-GAC-CCC-CTG-GAC-AGT-TCA-TTA-TCA-ACC-AAA-C | Point mutation of MAKPW-CL plasmid to MATPW-CL | Eurogentec |
