# Supplementary material for: Ribosomal stalling landscapes revealed by high-throughput inverse toeprinting of mRNA libraries
Source: Life Sci Alliance. 2018 Oct 9;1(5):e201800148. doi: 10.26508/lsa.201800148 (PMC6238534; doi:10.26508/lsa.201800148)
Supplement: Supplementary file 7 [file LSA-2018-00148_TableS7.docx]

**Supplementary Table S7 ­– Summary of NGS read processing**

| Sample | Reads before filtering | Outside region of interest | Contains long ‘A’ stretch | First codon not ATG | Q < 30 | Reads after filtering |
| --- | --- | --- | --- | --- | --- | --- |
| NNS_15_-NoAb1 | 2,877,735 | 1,679,246 | 143 | 14,283 | 173,880 | 1,010,183 |
| NNS_15_-NoAb2 | 5,284,297 | 2,703,215 | 294 | 16,871 | 155,256 | 2,408,661 |
| NNS_15_-Ery1 | 4,949,363 | 2,654,430 | 162 | 15,190 | 104,646 | 2,174,935 |
| NNS_15_-Ery2 | 6,780,268 | 3,639,577 | 230 | 22,590 | 189,211 | 2,928,660 |
| NNS_15_-NoAb1 +EF-P | 10,765,416 | 6,024,995 | 470 | 51,040 | 790,792 | 3,898,119 |
| NNS_15_-Ery1 +EF-P | 4,515,200 | 2,062,760 | 92 | 22,025 | 411,639 | 2,018,684 |
| NNS_15_ Library | 1,200,518 | 108,885 | 0 | 1,590 | 211,260 | 878,783 |
| ErmBL-Ery | 1,162,143 | 281,581 | 0 | 3,498 | 157,270 | 719,776 |
| ErmBL-Ole | 1,041,342 | 237,584 | 0 | 3,205 | 144,784 | 655,769 |
| ErmBL Library | 1,402,702 | 90,215 | 0 | 1,740 | 37,054 | 1,273,693 |
