# Supplementary material for: Ribosomal stalling landscapes revealed by high-throughput inverse toeprinting of mRNA libraries
Source: Life Sci Alliance. 2018 Oct 9;1(5):e201800148. doi: 10.26508/lsa.201800148 (PMC6238534; doi:10.26508/lsa.201800148)
Supplement: Supplementary file 8 [file LSA-2018-00148_TableS8.docx]

**Supplementary Table S8 – Nucleotide sequences for arrest peptides used in this study**

| Arrest sequence | Coding sequence |
| --- | --- |
| *ermAL* | ATGTGCACCAGTATCGCAGTAGTAGAA |
| *ermBL* | ATGTTGGTATTCCAAATGCGTAATGTAGATAAA |
| *ermCL* | ATGGGCATTTTTAGTATTTTTGTAATCAGC |
| *ermDL* | ATGACACACTCAATGAGACTTCGT |
| *secM_150-166_* | ATGTTCAGCACGCCCGTCTGGATAAGCCAGGCGCAAGGCATCCGTGCTGGCCCT |
| *tnaC_12-24_UAA_25_* | ATGTGGTTCAATATTGACAACAAAATTGTCGATCACCGCCCTTAA |
